# Gene screening of colorectal cancers via network analysis 

**Published:** 2019

**Authors:** Vahid Mansouri, Mostafa Rezaei Tavirani, Sina Rezaei Tavirani

**Affiliations:** 1 *Proteomics Research Center, Faculty of Paramedical Sciences, Shahid Beheshti University of Medical Sciences, Tehran, Iran*; 2 *Proteomics Research Center, Shahid Beheshti University of Medical Sciences, Tehran, Iran*

**Keywords:** Colorectal cancer, Biomarker, Gene

## Abstract

**Aim:**

Identifying crucial genes related to colorectal cancers via protein-protein interaction (PPI) network analysis is the aim of this study.

**Background:**

colorectal cancer as major reason of mortality is evaluated by genetic and proteomic approaches to find suitable biomarkers. Chromosomal instability plays crucial role in CRC. Expression change of large numbers of genes is reported.

**Methods:**

Differentially expressed genes related to CRCs which obtained from different proteomic methods were extracted from a review article of Paula Álvarez-Chaver *et al*. The genes interacted by Cytoscape software via STRING database. The central nodes determined and were enriched for biological terms by ClueGO. Action map for central genes was illustrated by CluePedia. The critical genes in CRC were introduced.

**Results:**

Among 123 query genes, 114 one recognized by software and were included in the network. SRC, EGFR, PCNA, IL8, CTNNB1, TIMP1, CDH1, and HSPD1 were determined as central genes. After gene ontology analysis SRC, EGFR, and CDH1 were identified as critical genes related to CRC.

**Conclusion:**

It seems that SRC, EGFR, and CDH1 and the related pathways are possible biomarkers for CRC.

## Introduction

 Colorectal cancer (CRC) is known as a major reason of morbidity and mortality by cancers (1). There are evidence that early detection and treatment of CRCs are correlated to decrement of mortality rate (2). Serious efforts by researchers are opened new fields in personalized cancer medicine about CRCs to find precise diagnostic and therapeutic methods based on genetic milieu (3). It is reported that clinical behavior of CRCs is caused by different molecular level especially chromosomal instability (4). Deregulation of many genes such as EGFR and cyclooxygenase 2 in CRC patients is confirmed (5, 6). It is reported that carcinoembryonic antigen (CEA) and carbohydrate antigen 19-9 (CA 19-9) levels are elevated in CRCs (7). Since expression change of large numbers of genes is associated with diseases, introducing limited numbers of them as diagnostic or therapeutic biomarkers is aim of numerous researchers. Using screening methods and validation tests can provide useful tools to find effective biomarkers (8, 9). 

PPI network analysis is an efficient method to screen large numbers of genes based on interaction between them. In this approach the query genes are interacted in and interactome (10) in two style; scale free network and non-scale free network. Usually gene networks are scale free and the elements of network can be differentiated based on centrality parameters such as degree, betweenness centrality, closeness centrality, and stress (11, 12). The nodes of network which have high numbers of connections are known as hubs and play crucial role in network integrity. Bottleneck nodes are characterized by high value betweenness. Common hubs and bottlenecks which are called hub-bottlenecks are critical elements of PPI network (13). Based on network analysis, it is possible to find common pathway between two diseases. In this regard, investigation indicates that there are common molecular features between colon and breast cancers (14). Many differentially expressed genes in CRC patients are introduced, screen of them to find crucial ones is aim of this study. 

## Methods

Paula Álvarez-Chaver *et al.* (2014) published a review about CRC biomarkers that were discovered by different tolls of proteomics from various biological samples (15). In the present study the 123 introduced biomarkers in this report are used to screen via PPI network analysis (see table S1). The genes included in interacted unit by Cytoscape software version 3.6.0 (16) and STRING database (17). The constructed network was analyzed by Network analyzer and the hub-bottlenecks were determined based on degree values and betweenness centrality. Common hub-bottlenecks and top nodes based on closeness centrality and stress were identified as central nodes. Action map including activation, inhibition, and expression were illustrated for the central nodes by CluePedia (18). Kapa score is considered as default value in CluePedia. Biological terms related to the central genes were determined by ClueGO (19) and were clustered. P value less than 0.05 was considered. Function and role of central nodes in CRC were discussed and interpreted in details. 

## Results

**Figure 1:**
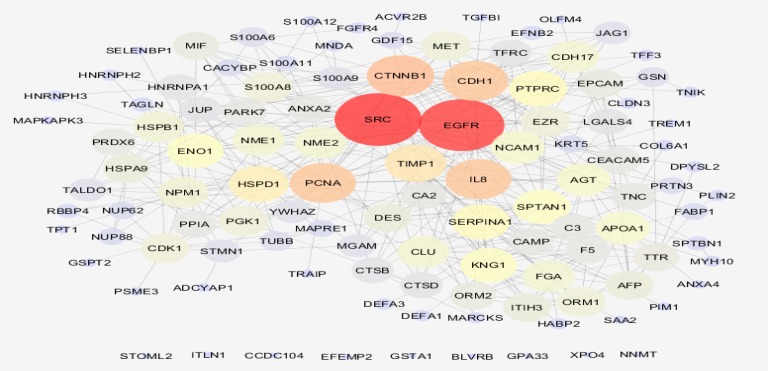
PPI network of colon cancer including 9 isolated nodes and a main connected component

**Figure 2:**
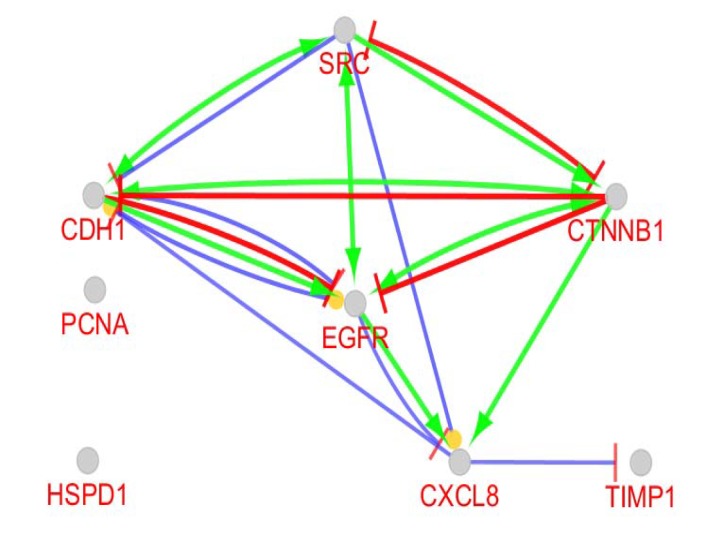
Action map including activation, inhibition, and expression relative to the 8 hub-bottlenecks. Blue, green, and red colors refer to expression, activation, and inhibition actions. Kapa score is considered as default value in CluePedia

PPI network including 114 DEGs was constructed (see figure 1). The network contains 9 isolated nodes and a main connected component (105 nodes and 409 edges). Top 10% of nodes based on degree value including SRC, EGFR, CTNNB1, CDH1, IL8, PCNA, TIMP1, HSPD1, PTPRC, and SERPINA1 were selected as hubs. Top 10 nodes regarding betweenness centrality contain SRC, EGFR, PCNA, IL8, CTNNB1, TIMP1, HSPB1, CDH1, SPTAN1, and HSPD1 were determined as bottlenecks. Common hubs and bottlenecks including SRC, EGFR, PCNA, IL8, CTNNB1, TIMP1, CDH1, and HSPD1 were identified as hub-bottlenecks. All hub-bottlenecks were included in top nodes based on closeness centrality and stress (see table 1). Action map including expression, activation, and inhibition relative to the hub-bottlenecks is illustrated in the figure 2. 

**Table 1:** Hub-bottlenecks of colorectal cancer network based on data from proteomic reports. These nodes are included in top nodes based on closeness centrality and stress

R	name	description	D	BC	CC	Stress
1	SRC	v-src sarcoma (Schmidt-Ruppin A-2) viral oncogene homolog (avian)	36	0.20	0.58	9508
2	EGFR	epidermal growth factor receptor	35	0.16	0.57	7840
3	CTNNB1	catenin (cadherin-associated protein), beta 1, 88kDa	23	0.07	0.49	3608
4	CDH1	cadherin 1, type 1, E-cadherin (epithelial)	22	0.06	0.49	2880
5	IL8	interleukin 8	22	0.09	0.51	5086
6	PCNA	proliferating cell nuclear antigen	22	0.09	0.51	4782
7	TIMP1	TIMP metallopeptidase inhibitor 1	19	0.06	0.49	3924
8	HSPD1	heat shock 60kDa protein 1 (chaperonin)	18	0.05	0.46	2548

**Figure 3:**
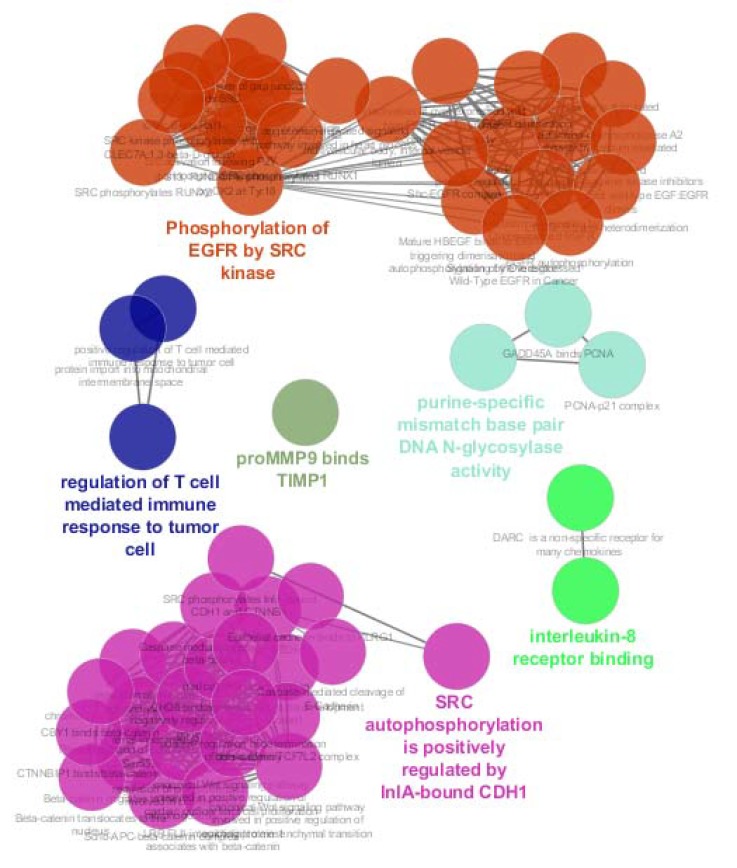
Biological terms relative to the 8 hub-bottlenecks. The colored terms are names of groups. Grouping p value and p value ≤ 0.05 were considered

**Figure 4:**
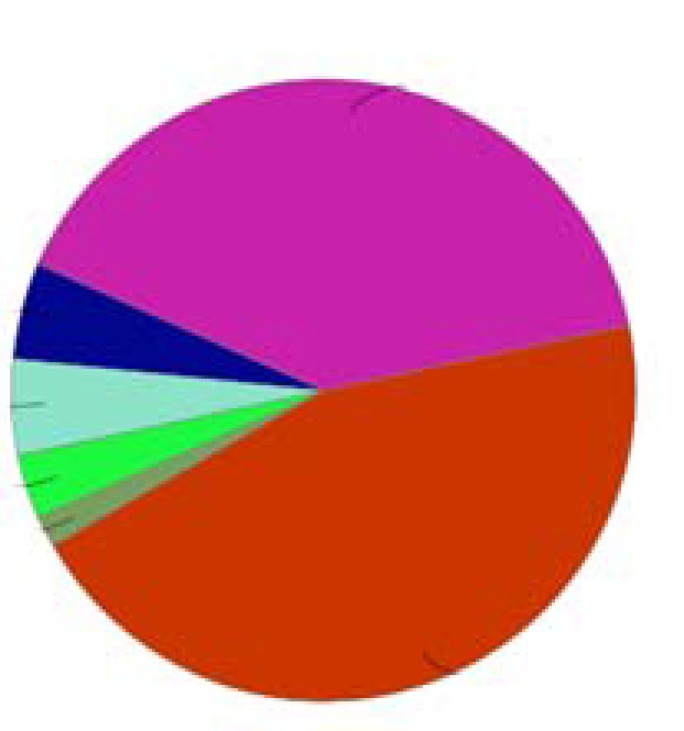
Six clusters of biological term relative to the 8 central nodes are shown. Names of groups correspond to the group colors are written in the foot of figure.

Further investigation about biological terms related to the hub-bottlenecks is shown in the figure 3. Numbers of 60 terms are identified which are attributed to the 8 central nodes. The determined terms are clustered in 6 classes including SRC auto phosphorylation is positively regulated by InlA-bound CDH1, Phosphorylation of EGFR by SRC kinase, proMMP9 binds TIMP1, interleukin-8 receptor binding, purine-specific mismatch base pair DNA N-glycosylase activity, and regulation of T cell mediated immune response to tumor cell. Graphical presentation of 6 groups is shown in the figure 4.

## Discussion

Introducing 123 cancer markers is an opportunity to find the best ones. It is possible to screen and select the crucial individuals that are prominent roles in onset and progress of CRC. Numbers of 114 genes among 123 ones (about 93%) were recognized by STRING database. The network was included 9 isolated nodes (about 8%) and a main connected component contains 105 nodes and 409 edges. Numbers of 8 central nodes were introduced. In many studies hubs or bottlenecks are introduced as central node, however it is reported that hub-bottlenecks are crucial genes or proteins.

In this investigation the hub-bottlenecks which were top nodes based on both closeness centrality and stress were introduced as central nodes. This procedure indicates that the selected central nodes are potent central nodes and with high rate of confidence are the critical nodes of the studied network. Action map analysis revealed that except PCNA and HSPD1 the other central nodes are participated in the action map. As it is shown in the table 1, these two genes are in the row positions 6 and 8, therefore the weaker hubs are among the identified central nodes. Also, it is clear that row 7 is occupied by TIMP1. This gene is an individual among interacted genes in action map which only is regulated by CXCL8 (IL8) and has no regulatory effect on the other genes. Ignoring Timp1, the 5 remained genes including SRC, EGFR, IL8, CTNNB1, and CDH1 are highly connected to each other. As it is depicted in the figures 3 and 4, numbers of 60 biological terms related to the 8 central nodes are identified which are groups in the 6 clusters. Two clusters including “SRC autophosphorylation is positively regulated by InlA-bound CDH1” and “Phosphorylation of EGFR by SRC kinase” contain 51 terms or 85% all biological terms. It seems that these two classes of biological terms are the core of terms related to the central nodes. As it is appeared both groups are concerned by SRC, CDH1, and EGFR genes. As it is shown in the action map IL8 (CXCL8) has no inhibition link with the other elements of network and it is activated by CTNNB1 and EGFR. IL8 also has no activator activity on the other nodes of the network. CTNNB1 is the third elements in the table 1. Comparison between CTNNB1 and the top 2 central nodes shows considerable differences between them based on degree vale and the other centrality parameters. Finally based on these findings, SCR, EGFR, and CDH1 can be considering as critical genes which are involved in CRC. In following part, functional roles of these three critical genes in body and cancers are discussed:

SRC is an important element in signal transduction pathways in normal and cancerous cells. Different pathways are identified that are related to SRC such as cell survival, adhesion, division and motility (20). 

□ SRC auto phosphorylation is positively regulated by InlA-bound CDH.

□ Phosphorylation of EGFR by SRC kinase.

□ proMMP9 binds TIMP1.

□ interleukin-8 receptor binding.

□ purine-specific mismatch base pair DNA N-glycosylase activity.

□ Regulation of T cell mediated immune response to tumor cell.

Investigations indicate that SRC is up-regulated in various cancers such as brain, colon, pancreas, and breast cancers. High level of SRC protein is found in cancerous tissue relative to adjacent normal tissue. It is reported that there is correlation between SRC level and malignancy process progression (21). Bosutinib, dasatinib, saracatinib, and KX01 are introduced as SRC inhibitors (22). EGFR the other crucial genes codes the transmembrane tyrosine kinase which activated by EGF family ligand. EGFR activation leads to activation of MAPK signaling pathway that is responsible for cell proliferation promotion (23). Deregulation of EGFR in wide varieties of tumors is reported. Since inhibition of EGFR in chemotherapy is an important aim, two classes of EGFR inhibitors are identified including monoclonal antibodies such as cetuximab (Erbitux) which target extracellular domain of EGFR and the other class of small molecule tyrosine kinase inhibitors as like erlotinib and gefitinib (24).

CDH1 the third crucial protein in this analysis is a critical agent in cell-cell adhesion. CDH1 is known as tumor suppressor and its down regulation in several cancers such as gastric cancer is reported (25). There is evidence that CDH1 gene hyper methylation is associated with CRC (26). As it is reported CDH1 and CDH13 methylation in serum can be considered as cervical cancer markers (27).

It seems that SRC, EGFR, and CDH1 are suitable biomarker candidates for CRC. However, quantity profiling of the elements of this panel may be useful tool in diagnosis and follow of CRC patients. Besides diagnostic features, these genes may be a appreciate drug targets in CRC disease.
